# Synthesis and biological evaluation of aryl-oxadiazoles as inhibitors of *Mycobacterium tuberculosis*

**DOI:** 10.1016/j.bmcl.2018.04.028

**Published:** 2018-06-01

**Authors:** Maria Angeles Martinez-Grau, Isabel C. Gonzalez Valcarcel, Julie V. Early, Richard Klaus Gessner, Candice Soares de Melo, Eva Maria Martin de la Nava, Aaron Korkegian, Yulia Ovechkina, Lindsay Flint, Anisa Gravelle, Jeff W. Cramer, Prashant V. Desai, Leslie J. Street, Joshua Odingo, Thierry Masquelin, Kelly Chibale, Tanya Parish

**Affiliations:** a Lilly Research Laboratories, Eli Lilly and Company, Avda. de la Industria 30, 28108-Alcobendas, Madrid, Spain; b Lilly Research Laboratories, Eli Lilly and Company, Indianapolis, IN 46285, USA; c TB Discovery Research, Infectious Disease Research Institute, 1616 Eastlake Ave E, Suite 400, Seattle, WA 98102, United States; d Drug Discovery and Development Centre (H3D), Department of Chemistry, University of Cape Town, Rondebosch 7701, South Africa; e South African Medical Research Council Drug Discovery and Development Research Unit, Department of Chemistry, University of Cape Town, Rondebosch 7701, South Africa; f Institute of Infectious Disease and Molecular Medicine, University of Cape Town, Rondebosch 7701, South Africa

**Keywords:** Mycobacterium tuberculosis, Oxadiazoles, Phenotypic screening, Antibacterial

## Abstract

Despite increased research efforts to find new treatments for tuberculosis in recent decades, compounds with novel mechanisms of action are still required. We previously identified a series of novel aryl-oxadiazoles with anti-tubercular activity specific for bacteria using butyrate as a carbon source. We explored the structure activity relationship of this series. Structural modifications were performed in all domains to improve potency and physico-chemical properties. A number of compounds displayed sub-micromolar activity against *M. tuberculosis* utilizing butyrate, but not glucose as the carbon source. Compounds showed no or low cytotoxicity against eukaryotic cells. Three compounds were profiled in mouse pharmacokinetic studies. Plasma clearance was low to moderate but oral exposure suggested solubility-limited drug absorption in addition to first pass metabolism. The presence of a basic nitrogen in the linker slightly increased solubility, and salt formation optimized aqueous solubility. Our findings suggest that the 1,3,4-oxadiazoles are useful tools and warrant further investigation.

## Highlights

• We conducted SAR for the aryl-oxadiazole series with anti-bacterial activity against *Mycobacterium tuberculosis.*
• Improved compounds have sub-micromolar butyrate-specific activity.
• Compounds are not cytotoxic against eukaryotic cells.
• A basic nitrogen in the linker and salt formation improved aqueous solubility.

## Graphical abstract

**Figure:**
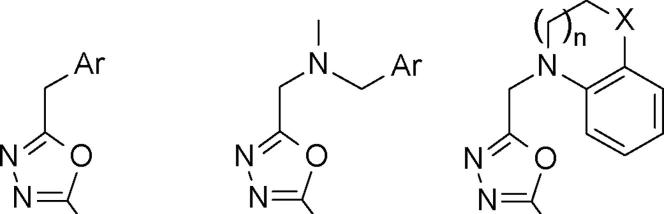

Tuberculosis is the leading cause of death from infectious disease.^1^ Although the number of tuberculosis cases decreased during the 20th century, the emergence of HIV and the incidence of multiple-drug resistance have increased the difficulty of treating many new cases.^1^, ^2^ Despite efforts to improve the outcome of tuberculosis care, the discovery of new antibiotics against the causative agent *Mycobacterium tuberculosis* has been insufficient to eradicate the disease.^3^ New and more effective drugs with novel mechanisms of action are required to shorten treatment, improve patient adherence, and reduce the appearance of resistance.

*Mycobacterium tuberculosis* can adapt metabolically to host environments and can catabolize multiple carbon sources simultaneously.^4^ Fatty acids are the major carbon source available during infection,^5^ although carbohydrates, lipids, and carbon dioxide can also be utilized as carbon sources.^6^

We recently reported the identification of a family of oxadiazoles **1**–**5** (Fig. 1)^7^ from a whole cell screen against *M. tuberculosis* using butyrate as the carbon source. The compounds were active in medium containing butyrate, but not glucose and lacked mammalian cytotoxicity.^7^, ^8^ The lack of cytotoxicity and the low molecular weight prompted us to undertake structure activity relationship (SAR) investigations around this series.

**Fig. 1:**
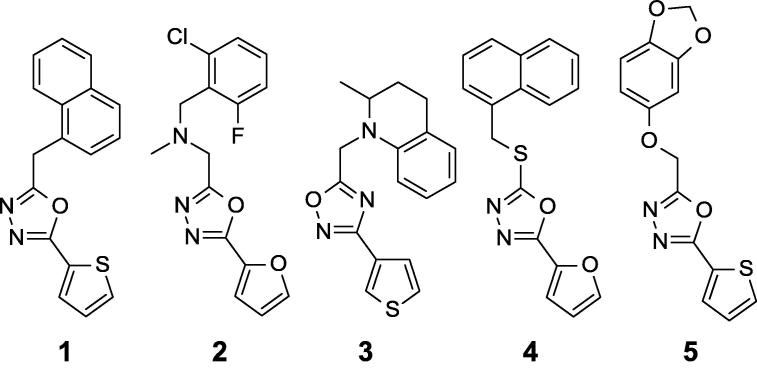
Oxadiazoles previously identified from whole cell screening against *Mycobacterium tuberculosis,* adapted from Early et al.^7^

Aryl-oxadiazoles, the common structural motif in compounds **1**–**5**, have been widely applied in medicinal chemistry for the development of new drugs. Compounds containing the 1,2,4- and 1,3,4-oxadiazole motif have been evaluated against a broad spectrum of pharmacological activities, with special attention to their properties as antimicrobial and antitubercular agents.^9^, ^10^, ^11^, ^12^

Synthetic methods for the preparation of differently functionalized 1,3,4-oxadiazoles have been recently reviewed.^13^ Compound 2 was resynthesized and compounds **13**–**18** and **24**–**41** were made in three steps by the method previously published for making compound **2**, starting from the corresponding hydrazide and then reacting the intermediate chloride with the appropriate secondary amine.^7^ Compounds **6**–**9** were prepared according to the representative procedure exemplified in Scheme 1 for compound **8**. Hydrazide **8a** was coupled with carboxylic acid **8b** using EDC and HOBt to obtain the intermediate **8c**. Cyclodehydration of semicarbazide **8c** by refluxing with phosphoryl chloride yielded compound **8**.

**Scheme 1:**
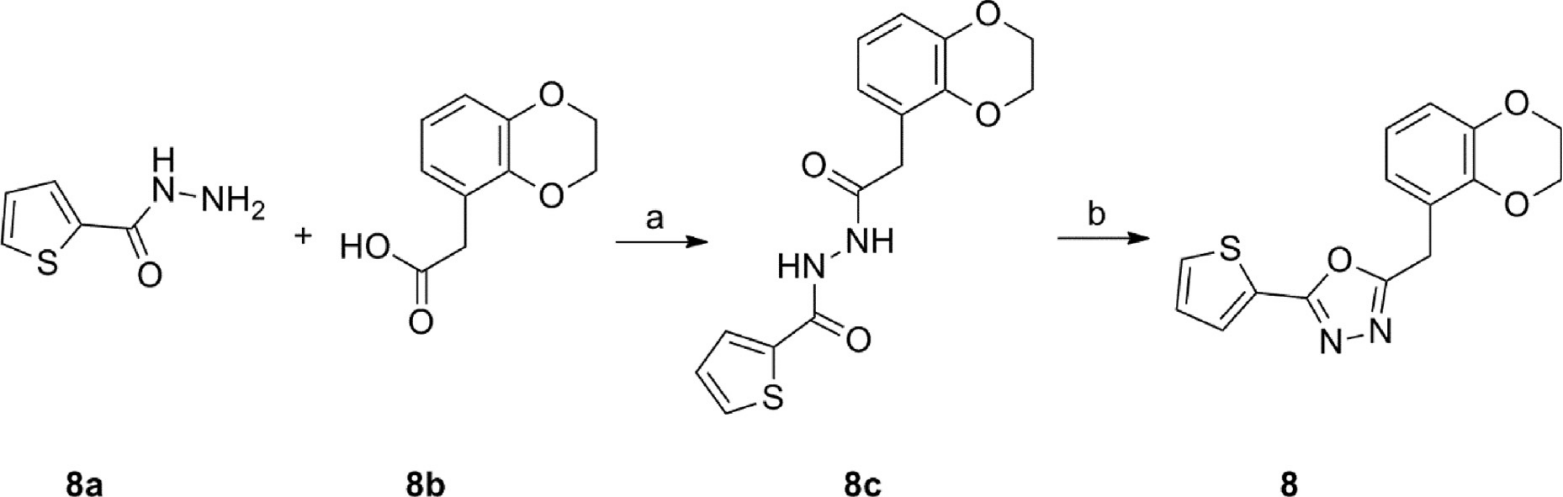
Synthesis of compound **8**. Reagents and conditions: (a) EDC, HOBt, DMF; (b) POCl_3_, 110 °C.

In order to prepare thiadiazole **10**, reaction of furan-2-carbohydrazide **10a** with chloroacetyl chloride in the presence of *N*-methylmorpholine produced the intermediate acylsemicarbazide **10b** (Scheme 2). Acylsemicarbazide **10b** was refluxed with Lawesson’s reagent in THF to obtain the intermediate chloride **10c**. Chloride replacement by 2-chloro-6-fluorobenzylamine at reflux in the presence of DIPEA and sodium iodide generated the secondary amine **10d** which was treated with sodium hydride and methyl iodide to give compound **10**. Compounds **11**, **12** and **19** were prepared from the corresponding oxadiazole analogue to chloride **10c** by reaction with the appropriate primary amine followed by methylation.

**Scheme 2:**
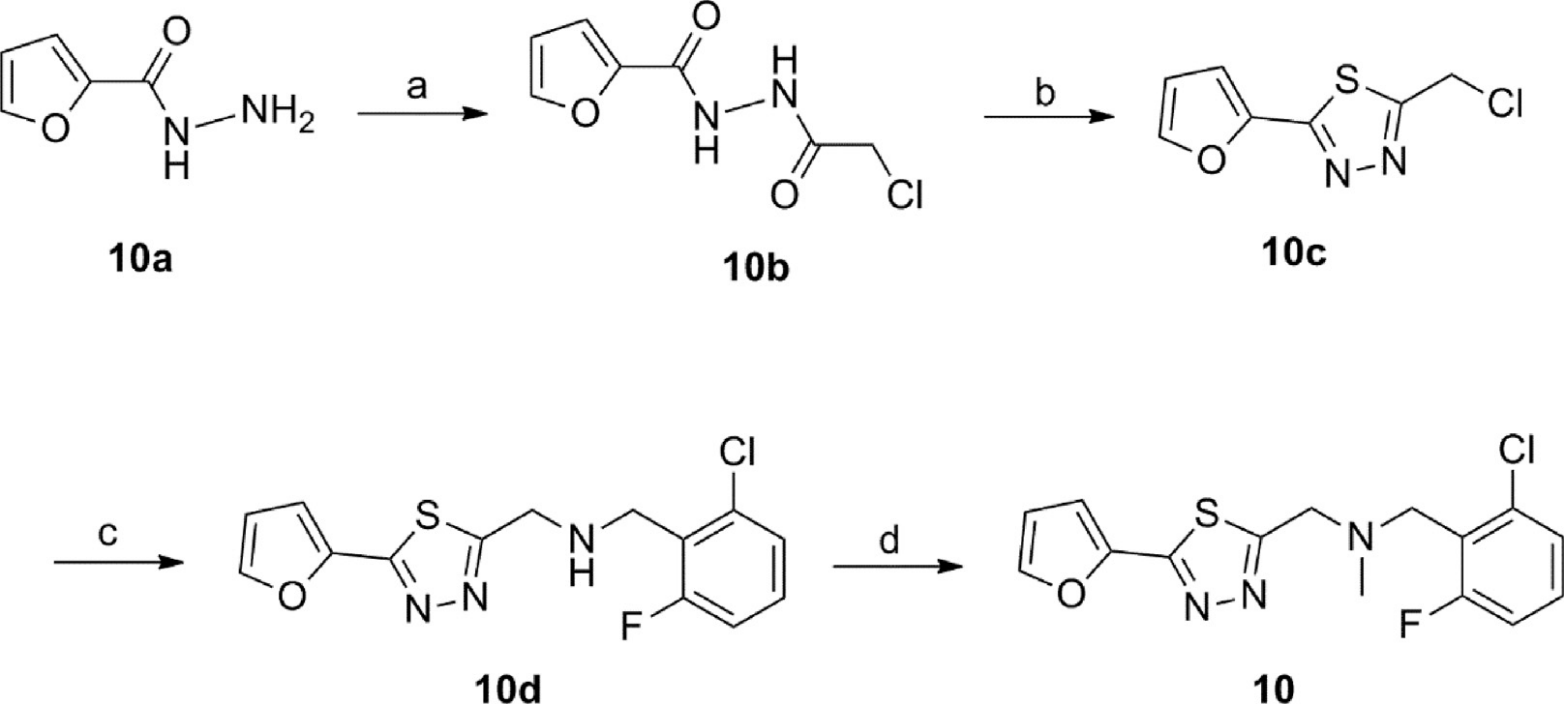
Synthesis of compound **10**. Reagents and conditions: (a) *N*-methylmorpholine, chloroacetyl chloride, CH_2_Cl_2_; (b) Lawesson’s reagent, THF, reflux; (c) 2-Chloro-6-fluorobenzylamine, DIPEA, NaI, CH_3_CN, reflux; (d) MeI, NaH, DMF.

The synthesis of the oxadiazole **21** containing an ether group in the linker was achieved by cyclization of acylsemicarbazide **10b** using phosphoryl chloride to create **21a** followed by the reaction of the intermediate **21a** with (2-chloro-6-fluorophenyl)methanol (Scheme 3). Amide **22** was prepared from chloride **21a** in two steps, via **22a**. Substitution of chloride **21a** by methylamine followed by amide formation using 2-chloro-6-fluorobenzoic acid in the presence of HATU and triethylamine provided the amide **22** (Scheme 3). Compound **20** was prepared by reaction of intermediate **21a** with 2-chloro-6-fluorobenzylamine using DIPEA and sodium iodide.

**Scheme 3:**
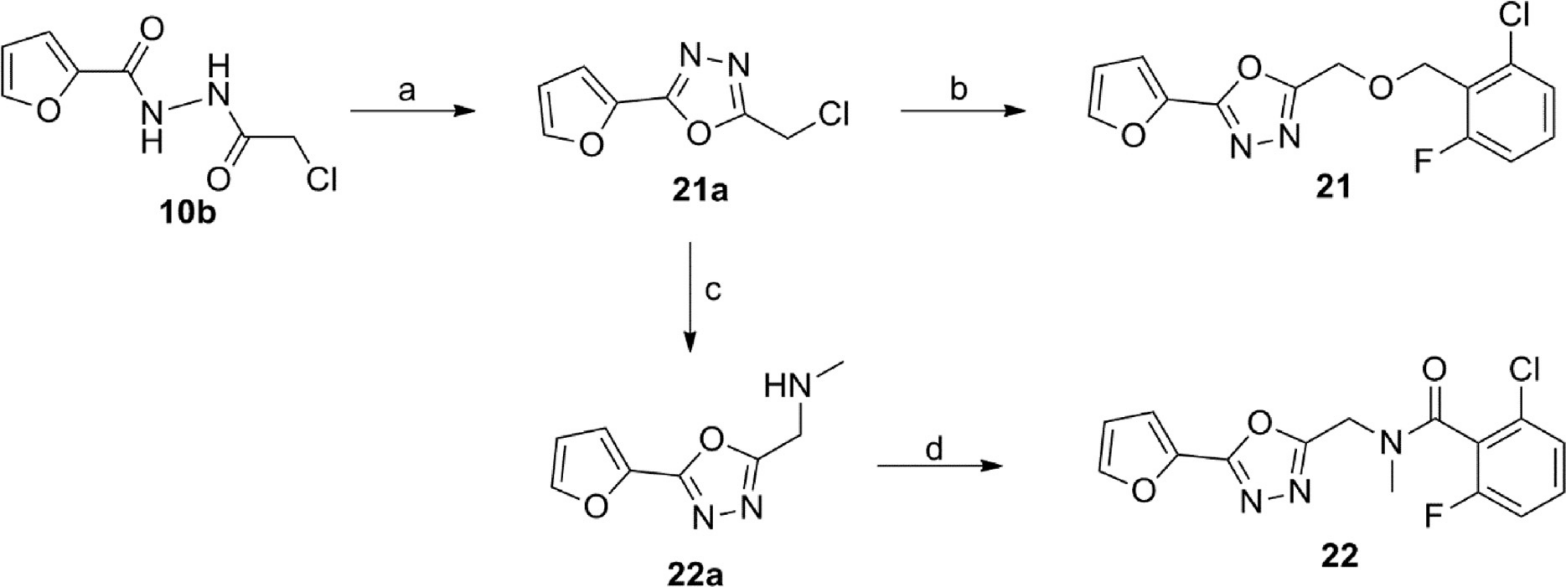
Synthesis of compounds **21** and **22**. Reagents and conditions: (a) phosphoryl chloride; (b) (2-Chloro-6-fluorophenyl)methanol, NaH, THF; (c) MeNH_2_, NaI, CH_3_CN, reflux; (d) 2-Chloro-6-fluorobenzoic acid, HATU, Et_3_N, DMF.

The synthesis of compound **23** utilized the three-step route shown in Scheme 4. Commercially available 1,3,4-oxadiazol-2-one **23a** was refluxed with 2-chloro-6-fluoro-phenethylamine **23b** in ethanol to obtain intermediate **23c** that was cyclized by heating with phosphoryl chloride. Methylation of secondary amine **23d** using methyl iodide in the presence of sodium hydride in DMF afforded compound **23**.

**Scheme 4:**
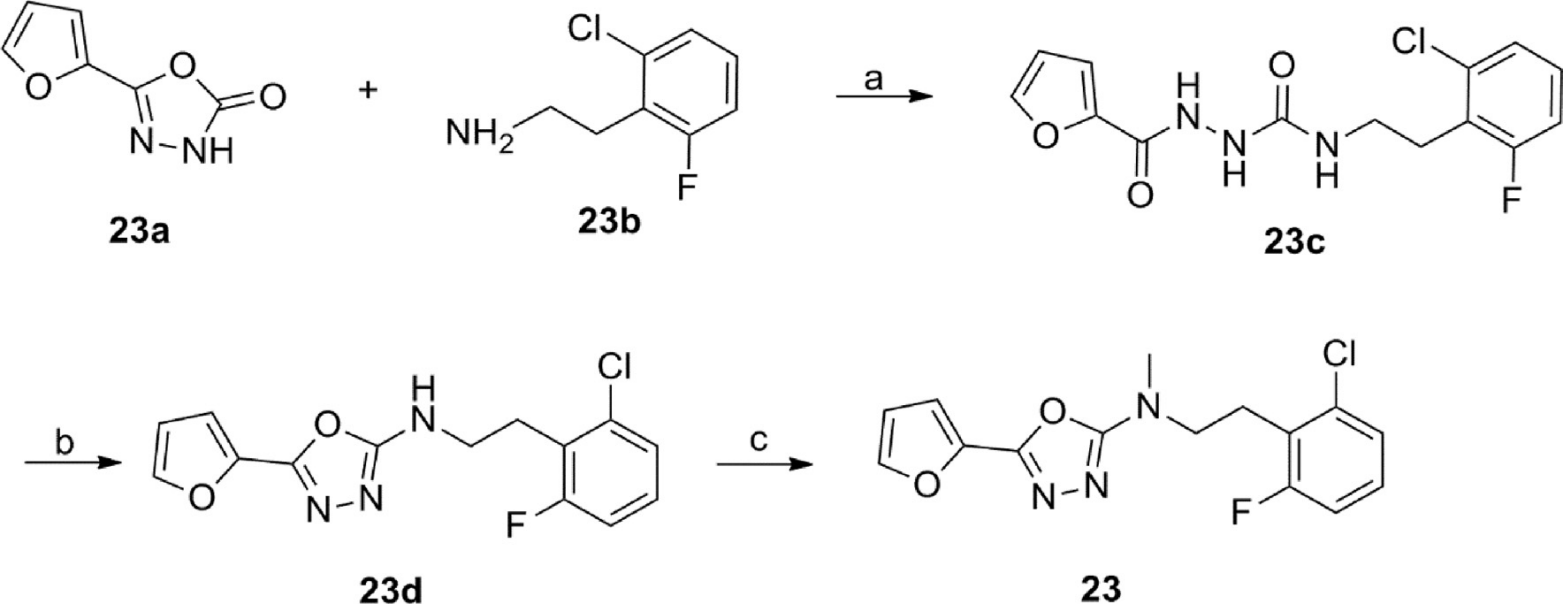
Synthesis of compound **23**. Reagents and conditions: (a) EtOH, 100 °C; (b) POCl_3_, 110 °C; (c) MeI, NaH, DMF.

We began by evaluating mouse microsomal metabolism and thermodynamic solubility for aryl-oxadiazole hits from the screen (Fig. 1, Table 1), and learned that these compounds were all highly metabolized, and solubility varied. We then focused our attention on the most potent hit from the screen, compound **1** (previously reported MIC was 0.4 ± 0.1 µM in butyrate medium)^7^ and synthesized compounds **6**–**9** (Fig. 2) to answer specific structure-activity questions. In order to compare 2-thienyl- with 2-furyl-oxadiazole, the thiophene ring in compound **1** was replaced with furan **6** (Fig. 2, Table 2). These two compounds have comparable activity in butyrate medium (7H9-Ty-BT) (Table 2). Replacements for 1-naphthyl in compound **1** were also examined, detecting that 2-naphthyl **7** was less potent while 5-benzodioxine **8** or phenyl **9** retained activity Compounds **6**–**9** showed neither activity under glucose conditions (7H9-Tw-OADC) nor cytotoxicity in Vero cells (Table 2).

**Fig. 2:**
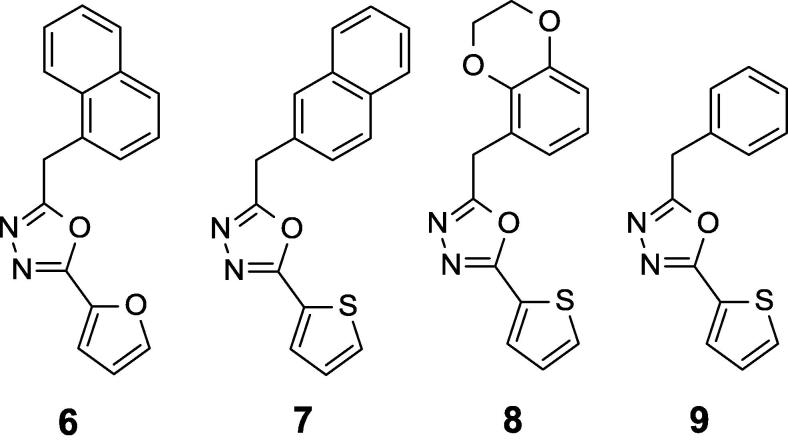
SAR around compound **1**.

**Table 1:** Mouse microsomal metabolism, unbound intrinsic clearance^14^ and aqueous solubility for aryl-oxadiazoles from the screen.

Cpd	Mouse microsome (% metabolized in 30 min)	Mouse microsome unbound intrinsic clearance (ml/min/kg)	Aqueous solubility at pH 7.4 (mg/mL)	clogP^a^
**1**	99	3310	<0.001	3.9
**2**	99	1350	0.077	2.5
**3**	65	1910	0.005	4.4
**4**	95	1680	<0.001	3.7
**5**	76	332	0.018	2.3

a clogP values are calculated from BioByte software.

**Table 2:** Biological activity for compounds exploring SAR on the most potent screening hit.

Cpd	7H9-Ty-BT^a^^,^^b^ MIC (μM)	7H9-Tw-OADC^a^^,^^c^ MIC (μM)	Vero IC_50_ (μM)	Mouse microsome (% metabolized)	Aqueous solubility at pH 7.4 (mg/mL)	clogP^d^
**6**	1.1 ± 0.9	>20	>100	100	<0.001	3.1
**7**	5.7 ± 0.2	>20	>100	100	<0.001	3.9
**8**	0.8 ± 0.2	>20	>100	99	0.077	2.6
**9**	1.1 ± 0.2	>20	79	99	0.031	2.7

a Results are average ± standard deviation for at least 2 runs.

b Growth medium with butyrate as the primary carbon source.

c Growth medium with glucose as the primary carbon source.

d clogP values are calculated from BioByte software.

Compound **1** (Fig. 1, Table 1) and the analogs **6**–**9** (Table 2) suffered from very high microsomal metabolism. Although aqueous solubility was low for the most lipophilic compounds **1**, **3**, **4**, **6** and **7**, a trend to improve solubility was observed when lowering calculated logP (clogP), as in compounds **2**, **5**, **8** and **9**.

The focus of SAR evaluation became improving solubility and metabolic stability while maintaining potency. With this goal, we explored the SAR around compound **2** (Fig. 1), which had a previously reported MIC of 0.8 ± 0.3 µM in butyrate medium^7^ based on the improved solubility with respect to compounds **1**, **3**–**5**. The 1,3,4-thiadiazole **10** (Fig. 3, Table 3) was slightly less potent and less soluble than the 1,3,4-oxadiazole (**2**), so we continued the SAR evaluation using 1,3,4-oxadiazole as the heterocyclic core. Replacement of the furan ring in **2** by thiophene **11** or phenyl **12** led to equipotent compounds with similar solubility. The lack of both substituents at the *ortho*-positions, as in compounds **13**–**14**, or the presence of a single substituent, as in compound **15**, did not change the antimicrobial activity relative to compound **2**, suggesting that substitution at *ortho*-positions is not required for activity. Nevertheless, the lack of one or both substituents at the *ortho*-positions decreased clogP and significantly increased solubility. Compound **16**, having a fluoro group at the 4-position of the benzyl substituent, had similar potency in comparison to compound **12**. However, the trifluoromethyl group at 4-position in compounds **17** and **18** substantially decreased the activity with respect to compounds **11** and **12** and negatively impacted on solubility. Replacement of the benzyl group with a cyclohexylmethyl group as in compound **19** was also well tolerated. With respect to the linker, the *N*-methyl group in compound **2** appears important for activity. Replacement by the secondary amine **20** or oxygen **21** decreased the activity, while the tertiary amide **22** resulted in complete loss of activity. As expected, the lower lipophilicity of compounds **20**–**22** compared to compound **2** was beneficial for solubility. Compound **23**, with the nitrogen directly attached to the oxadiazole ring, reduced activity and the absence of a basic nitrogen resulted in reduced solubility. As previously seen, there was no or low cytotoxicity against Vero cells for compounds **10**–**23** (Fig. 3, Table 3).

**Fig. 3:**
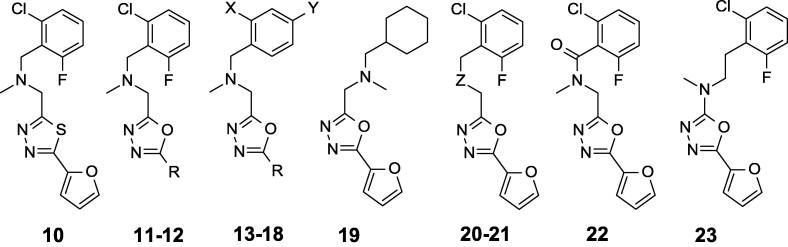
Analogues of compound **2**. SAR on aromatic domains and linker.

**Table 3:** Biological activity for compounds exploring SAR on aromatic domains and linker.

Cpd	R	X	Y	Z	7H9-Ty-BT^a^^,^^b^ MIC (μM)	7H9-Tw-OADC^a^^,^^c^ MIC (μM)	Vero IC_50_ (μM)	Mouse microsome (% metabolized)	Aqueous solubility at pH 7.4 (mg/mL)	clogP^d^
**10**					3.2 ± 0.5	>20	75	99	0.020	3.3
**11**	2-Thiophenyl				0.6 ± 0.2	>20	>100	100	0.12	3.1
**12**	Phenyl				0.4 ± 0.2	>20	>100	100	0.078	3.2
**13**	2-Thiophenyl	H	H		0.5 ± 0.1	>20	>100	98	0.42	2.2
**14**	2-Furanyl	H	H		0.6 ± 0.2	>20	>100	95	0.40	1.7
**15**	2-Furanyl	Cl	H		0.6 ± 0.2	>20	98	100	0.27	2.4
**16**	Phenyl	H	F		0.9 ± 0.03	>20	97	ND	ND	2.4
**17**	2-Thiophenyl	H	CF_3_		6.8 ± 2.4	>20	87	98	0.022	3.1
**18**	Phenyl	H	CF_3_		10.8 ± 3.3	>20	80	99	0.009	3.2
**19**					0.7 ± 0.5	>20	90	100	0.041	2.6
**20**				NH	5.5 ± 0.4	>20	>100	99	0.18	1.5
**21**				O	6.0 ± 0.4	>20	>100	100	0.15	2.1
**22**					>20	>20	>100	95	0.64	1.7
**23**					4.0 ± 0.6	>20	93	99	0.007	4.0

a Results are average ± standard deviation for at least 2 runs.

b Growth medium with butyrate as the primary carbon source.

c Growth medium with glucose as the primary carbon source.

d clogP values are calculated from BioByte software.

Compound **3** (Fig. 1), which had a previously reported MIC of 2.2 ± 0.4 µM in butyrate medium^7^, is a structurally constrained analogue containing a 1,2,4-oxadiazole core linked to a 3-thiophene instead of the 2-thiophene. Compound **24** (Fig. 4), a hybrid structure between compounds **3** and **5** containing the tetrahydroquinoline piece and the 1,3,4-oxadiazole core, was active (Table 4). A representative subset of tetrahydroquinoline compounds replacing the electron–rich thiophene by phenyl **25** or electron-deficient phenyls **26**, **27** was prepared. In this set, phenyl **25** failed to increase potency over **24** but, in contrast, 4-fluorophenyl **26** achieved a small improvement in activity and 4-trifluoromethylphenyl **27** retained potency. Interestingly, the tetrahydroisoquinoline regioisomers **28**–**31** were much less potent, showing that the site of fusion between the phenyl and the piperidine ring had a significant effect on activity. The clear preference for the tetrahydroquinoline orientation also indicates that the basicity of the nitrogen is not critical for maintaining potency although it influences solubility. 1,4-Benzoxazines **32**–**35** were less potent than the corresponding tetrahydroquinolines **24**–**27**. The size of the heterocyclic ring also impacted the activity. Indolines **36**–**37** lowered potency while isoindolines **38**–**39** lowered it even more, as expected based on the position of the nitrogen atom. When a nitrogen atom is part of the 2-phenylpyrrolidine ring as in **40**–**41**, compounds displayed minimal activity. As expected, compounds **24**–**41** were not cytotoxic and did not have activity against *M. tuberculosis* in medium containing glucose (Fig. 4, Table 4).

**Fig. 4:**
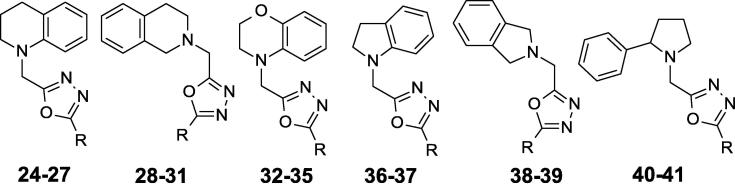
SAR around compound **24**, a hybrid structure between compounds **3** and **5**.

**Table 4:** Biological activity for compounds exploring SAR of the tetrahydroquinoline with the 1,3,4-oxadiazole.

Cpd	R	7H9-Ty-BT^a^^,^^b^ MIC (μM)	7H9-Tw-OADC^a^^,^^c^ MIC (μM)	Vero IC_50_ (μM)	Mouse microsome (% metabolized)	Aqueous solubility at pH 7.4 (mg/mL)	clogP^d^
**24**	2-Thiophenyl	0.8 ± 0.5	>20	>100	97	0.005	3.2
**25**	Phenyl	1.7 ± 0.1	>20	>100	100	0.003	3.5
**26**	4-F-Phenyl	0.2 ± 0.1	>20	>100	100	<0.002	3.5
**27**	4-CF_3_-Phenyl	0.7 ± 0.3	>20	>100	87	<0.002	4.2
**28**	2-Thiophenyl	3.7 ± 0.3	>20	>100	99	0.054	2.5
**29**	Phenyl	7.7 ± 0.8	>20	>100	99	0.051	2.6
**30**	4-F-Phenyl	4.7 ± 1.3	>20	>100	80	0.040	2.8
**31**	4-CF_3_-Phenyl	>20	>20	>100	ND	<0.002	3.5
**32**	2-Thiophenyl	1.8 ± 0.6	>20	>100	99	0.033	2.6
**33**	Phenyl	2.8 ± 1.7	>20	>100	99	0.028	2.7
**34**	4-F-Phenyl	1.8 ± 0.4	>20	>100	98	<0.002	2.9
**35**	4-CF_3_-Phenyl	8.3 ± 1.7	>20	85	95	<0.002	3.6
**36**	2-Thiophenyl	2.1 ± 0.1	>20	>100	99	0.038	2.8
**37**	Phenyl	2.3 ± 0.9	>20	>100	99	0.021	2.9
**38**	2-Thiophenyl	6.7 ± 1.7	>20	>100	99	0.012	2.0
**39**	Phenyl	7.5 ± 3.5	>20	>100	98	0.020	2.1
**40**	2-Thiophenyl	9.9 ± 2.1	>20	50	100	0.050	2.8
**41**	Phenyl	8.3 ± 1.4	>20	>100	99	0.034	2.9

a Results are average ± standard deviation for at least 2 runs.

b Growth medium with butyrate as the primary carbon source.

c Growth medium with glucose as the primary carbon source.

d clogP values are calculated from BioByte software.

All the compounds we synthesized suffered from poor microsomal stability in mice (Table 1, Table 2, Table 3, Table 4) and rats (data not shown). One of the first strategies to increase metabolic stability is to reduce the overall lipophilicity. However, a correlation between microsomal metabolism and clogP could not be observed as a few compounds that lowered calculated lipophilicity did not reduce oxidative metabolism (Table 3, Table 4). On the positive side, lipophilicity apparently plays a secondary role in potency because correlation between clogP and activity was not observed (Table 1, Table 2, Table 3, Table 4).

To further investigate the impact of microsomal metabolism on clearance, three of the structurally different screening hits were selected for mouse pharmacokinetic (PK) studies (Table 5). The PK evaluation of **1**, **2** and **3** was performed after intravenous (1 mg/kg) and oral (10 mg/kg) administration to mice. In order to confirm that clearance was due to oxidative metabolism, compound **2** was co-administered with 1-aminobenzotriazole (ABT), a non-selective cytochrome P450 inhibitor.^15^ Compound **2** was rapidly eliminated from the body, but clearance decreased significantly when co-administered with ABT, demonstrating that the high clearance was due to CYP-mediated metabolic oxidation. This result is consistent with the very low microsomal stability. Oral exposure was low and increased >10 fold when co-administered with ABT, proving that first pass metabolism limits exposure. Blood clearance for compound **1** was low but oral exposure was much lower than expected, suggesting solubility-limited drug absorption. Compound **3** had moderate clearance and the very low oral exposure may be due to first-pass metabolism and/or solubility-limited absorption.

**Table 5:** Mouse PK parameters for compounds **1**, **2**, and **3** after oral and intravenous administration.

		Intravenous administration (1 mg/kg)						Oral administration (10 mg/kg)			
Cpd	Clint,u (mL/min/kg)	AUC_0–24_ (ng·h/mL)	Cl (mL/min/kg)	AUC,u_0–24_ (ng·h/mL)	CL,u (mL/min/kg)	V_dss_ (L/kg)	t_1/2_ (h)	AUC_0–24_ (ng·h/mL)	Cmax (ng/ml)	Tmax (h)	Cmax,u (ng/ml)
1	3310	2900	19	32	1727	0.9	1.2	31	19	2.75	0.2
2	1350	300	56	37	455	1.1	0.3	48	31	0.25	3.8
2^a^	1350	2700	12	332	98	1.3	1.7	2300	888	0.66	109
3	1910	430	41	6	2733	6.3	2.9	10	9	0.25	0.1

Cl, clearance; Cl,u, Unbound clearance (Total Cl/unbound fraction in plasma); Vd, volume of distribution; t_1/2_, plasma elimination half-life; AUC, area under the curve AUC,u, unbound area under the curve (total AUC x unbound fraction in plasma). See supplementary information for the unbound fraction in plasma. For unbound concentration calculations, blood:plasma ratio was assumed to be 1.

a Compound **2** was co-administered with ABT (100 mg/kg, 1 h pre-dose), a non-selective CYP-P_450_ inhibitor.

We tried several things to improve aqueous solubility for this series. As a general trend, the presence of a basic nitrogen in the linker increased solubility, and solubility was further increased with additional reduction in lipophilicity as seen in Table 3. We also explored how compounds with basic character could benefit from forming salts to optimize solubility and biopharmaceutical properties. As expected, compound **2** as free base had much lower solubility (0.049 mg/mL at pH = 6 and 0.077 mg/mL at pH = 7.4) than the maleate salt (0.687 mg/mL at pH = 6 and 0.717 mg/mL at pH = 7.4). Based on this result, salt formation should be considered the preferred approach to increase aqueous solubility for this structural class containing a basic center.

In summary, we completed the SAR evaluation of a 1,3,4-oxadiazole series of compounds with activity against *M. tuberculosis*. Compounds had good anti-tubercular activity when tested against bacteria utilizing butyrate as a carbon source, but not with glucose as a carbon source. Although this series will require optimization of molecular properties to improve oral exposure, several 1,3,4-oxadiazoles are valuable tools that will facilitate further study including target identification.

## References

[1] World Health Organization. Global Tuberculosis Report 2017.

[2] Shah N.S., Wright A., Bai G.H. (2007). Worldwide emergence of extensively drug-resistant tuberculosis. Emerg Infect Dis.

[3] Koul A., Arnoult E., Lounis N., Guillemont J., Andries K. (2011). The challenge of new drug discovery for tuberculosis. Nature.

[4] de Carvalho L.P., Fischer S.M., Marrero J., Nathan C., Ehrt S., Rhee K.Y. (2010). Metabolomics of Mycobacterium tuberculosis reveals compartmentalized co-catabolism of carbon substrates. Chem Biol.

[5] Munoz-Elias E.J., McKinney J.D. (2006). Carbon metabolism of intracellular bacteria. Cell Microbiol.

[6] Shi L., Sohaskey C.D., Pheiffer C. (2010). Carbon flux rerouting during Mycobacterium tuberculosis growth arrest. Mol Microbiol.

[7] Early J.V., Casey A., Martinez-Grau M.A. (2016). Oxadiazoles Have Butyrate-Specific Conditional Activity against Mycobacterium tuberculosis. Antimicrob Agents Chemother.

[8] Ollinger J., Bailey M.A., Moraski G.C. (2013). A dual read-out assay to evaluate the potency of compounds active against Mycobacterium tuberculosis. PLoS ONE.

[9] Flipo M., Desroses M., Lecat-Guillet N. (2011). Ethionamide boosters: synthesis, biological activity, and structure-activity relationships of a series of 1,2,4-oxadiazole EthR inhibitors. J Med Chem.

[10] Spink E., Ding D., Peng Z. (2015). Structure-activity relationship for the oxadiazole class of antibiotics. J Med Chem.

[11] Suresh Kumar G.V., Rajendraprasad Y., Mallikarjuna B.P., Chandrashekar S.M., Kistayya C. (2010). Synthesis of some novel 2-substituted-5-[isopropylthiazole] clubbed 1,2,4-triazole and 1,3,4-oxadiazoles as potential antimicrobial and antitubercular agents. Eur J Med Chem.

[12] Karabanovich G., Zemanova J., Smutny T. (2016). Development of 3,5-dinitrobenzylsulfanyl-1,3,4-oxadiazoles and thiadiazoles as selective antitubercular agents active against replicating and nonreplicating Mycobacterium tuberculosis. J Med Chem.

[13] de Oliveira C.S., Lira B.F., Barbosa-Filho J.M., Lorenzo J.G., de Athayde-Filho P.F. (2012). Synthetic approaches and pharmacological activity of 1,3,4-oxadiazoles: a review of the literature from 2000–2012. Molecules.

[14] Austin R.P., Barton P., Cockroft S.L., Wenlock M.C., Riley R.J. (2002). The influence of nonspecific microsomal binding on apparent intrinsic clearance, and its prediction from physicochemical properties. Drug Metab Dispos.

[15] Watanabe A., Mayumi K., Nishimura K., Osaki H. (2016). In vivo use of the CYP inhibitor 1-aminobenzotriazole to increase long-term exposure in mice. Biopharm Drug Dispos.

