# Supplementary material for: Synthesis and biological evaluation of aryl-oxadiazoles as inhibitors of Mycobacterium tuberculosis
Source: Bioorg Med Chem Lett. 2018 Jun 1;28(10):1758–64. doi: 10.1016/j.bmcl.2018.04.028 (PMC5946847; doi:10.1016/j.bmcl.2018.04.028)
Supplement: Supplementary data 1 [file mmc1.docx]

***Supplementary Information for***

**Synthesis and biological evaluation of aryl-oxadiazoles as novel inhibitors of *Mycobacterium tuberculosis* from phenotypic screening**

**1. Materials and Instrumentation for Synthetic Procedures**

All reagents were obtained from commercial sources. Solvents were of analytical grade and used as purchased. The naming of the compounds was determined by using the IUPAC naming feature in MDL Accelrys® Draw version 4.1. Reactions were monitored using LC-MS or thin-layer chromatography (TLC) on Merck precoated aluminium silica gel sheets (Kieselgel 60 F-254). Flash chromatography was performed on ISCO Companion systems (UV=254 nm) using silica gel columns. Compounds were characterized by ^1^H NMR and LC-MS and had purity greater than 95%. ^1^H NMR spectra were recorded on Bruker spectrometers at the indicated frequency at room temperature. All chemical shifts are reported in ppm using TMS as the internal standard. NMR spectra were referenced to the residual solvent peak. Abbreviations s, d, dd, m, t and br denote singlet, doublet, double doublet, multiplet, triplet and broad, respectively. LC-MS was carried on a HPLC system. Electrospray mass spectrometry measurements, acquired in positive mode, were performed on a Mass Selective Detector quadrupole mass spectrometer interfaced to the HPLC system. The samples were analyzed by one of these methods:

Method 1: Used X-bridge C18 column (4.6 × 50 mm, 3.5 µ) operated to 50 °C. The mobile phase was a gradient of A (10 mM ammonium bicarbonate, pH=9) and B (acetonitrile), with B increasing linearly from 10% to 95% over 1.5 min. The flow rate was 1.8 mL/min and the UV wavelength was set to 214 nm. Ions between m/z 70-900 were captured after electrospray ionization of the eluted test sample.

Method 2: Used XTerra-C18 columns (2.1 × 50 mm, 3.5 µ) operated to 50 °C. The mobile phase was a gradient of A (10 mM ammonium bicarbonate, pH=9) and B (acetonitrile), with B increasing linearly from 10% to 100% over 3 min and then with 100% of solvent B for 0.75 min. The flow rate was 1.1 mL/min and the UV wavelength was set to 214 nm. Ions between m/z 100-800 were captured after electrospray ionization of the eluted test sample.

Method 3: Used Waters CNRP column (4.6 × 150 mm, 5.0 µ) operated to 50 °C. The mobile phase was a gradient of A (water with 0.05% trifluoroacetic acid) and B (acetonitrile with 0.05% trifluoroacetic acid), with B increasing linearly from 5% to 95% over 6 min. The flow rate was 1.5 mL/min and the UV wavelength was set to 214 nm. Ions between m/z 80-800 were captured after electrospray ionization of the eluted test sample.

**2. Synthetic Procedures**

**2.1. Synthetic Procedure for the Preparation of Compound 8**

***N*'-[2-(2,3-Dihydro-1,4-benzodioxin-5-yl)acetyl]thiophene-2-carbohydrazide (8c):** 1-(3-Dimethylaminopropyl)-3-ethylcarbodiimide hydrochloride (177 mg, 0.927 mmol, 1.5 equiv) and 1-hydroxybenzotriazole (125 mg, 0.927 mmol, 1.5 equiv) were added to a solution of thiophene-2-carbohydrazide (**8a**) (89 mg, 0.618 mmol, 1.0 equiv) and 2-(2,3-dihydro-1,4-benzodioxin-5-yl)acetic acid (**8b**) (0.12 g, 0.618 mmol, 1.0 equiv) in DMF (2 mL). The mixture was stirred at room temperature for 18 h. Water (20 mL) was added and the mixture was extracted twice with ethyl acetate (25 mL). The combined organic phase was washed with brine (20 mL), dried over Na_2_SO_4_, filtered and concentrated. The crude mixture was purified by flash chromatography (ISCO system, 20 g column) eluting with pentane/ethyl acetate (10:1 to 3:1 mixture) to give the title compound as a white solid (0.19 g, 97% yield). LC-MS (ESI) *m/z* 319 (M+H)^+^, RT=1.63 min, 98% purity, method 1.

**2-(2,3-Dihydro-1,4-benzodioxin-5-ylmethyl)-5-(2-thienyl)-1,3,4-oxadiazole (8):** A mixture of *N*'-[2-(2,3-dihydro-1,4-benzodioxin-5-yl)acetyl]thiophene-2-carbohydrazide (**8c**) (0.12 g, 0.377 mmol, 1.0 equiv) in phosphoryl chloride (5 mL) was heated at 110 ºC for 2 h. The excess of phosphoryl chloride was removed under vacuum. The residue was quenched with a mixture of ice-water (10 mL) and pH was adjusted to 12 with a 2N aqueous solution of sodium hydroxide. The mixture was extracted twice with ethyl acetate (50 mL). The combined organic phase was washed with brine (20 mL), dried over Na_2_SO_4_, filtered and concentrated. The crude mixture was purified by flash chromatography (ISCO system, 20 g column) eluting with pentane/ethyl acetate (4:1 mixture) to give the title compound as a yellow oil (56 mg, 49% yield). LC-MS (ESI) *m/z* 301 (M+H)^+^, RT=4.0 min, 99% purity, method 3. ^1^H NMR (400 MHz, CDCl_3_) δ 7.69 (d, 1H, *J*=3.6 Hz), 7.51 (d, 1H, *J*=4.8 Hz), 7.13 (dd, 1H, *J*=4.8, 3.6 Hz), 6.82 (m, 3H), 4.28 (m, 4H), 4.23 (s, 2H).

**2.2. Synthetic Procedures for the Preparation of Compound 10**

**N'-(2-Chloroacetyl)furan-2-carbohydrazide (10b):** *N*-methylmorpholine (3.3 mL, 29.92 mmol, 1.5 equiv) was added to a suspension of furan-2-carbohydrazide (**10a**) (2.5 g, 19.82 mmol, 1.0 equiv) in dichloromethane (25 mL). The mixture was cooled to 0 °C, then chloroacetyl chloride (1.9 mL, 23.91 mmol, 1.21 equiv) was added dropwise in 5 min. The mixture was stirred at room temperature for 3 h. Water (50 mL) was added and the mixture was stirred for 10 min. The solid was collected by filtration. The aqueous phase was extracted with a 3:1 mixture of dichloromethane/*iso*-propanol (40 mL). The combined organic phase was dried over Na_2_SO_4_, filtered and concentrated to obtain a solid. The solids were combined and dried under vacuum to give the title compound as a white solid (3.1 g, 77% yield). LC-MS (ESI) *m/z* (^35^Cl/^37^Cl) 203/205 (M+H)^+^, RT=0.84 min, 99% purity, method 1. ^1^H NMR (400 MHz, DMSO-d_6_) δ 10.41 (s, 1H), 10.31 (s, 1H), 7.91 (d, 1H, *J*=1.6 Hz), 7.23 (d, 1H, *J*=3.6 Hz), 6.67 (dd, 1H, *J*=3.6, 1.6 Hz), 4.18 (s, 2H).

**2-(Chloromethyl)-5-(2-furyl)-1,3,4-thiadiazole (10c):** Lawesson's reagent (1 g, 2.47 mmoles, 1.0 equiv) was added to a solution of *N*'-(2-chloroacetyl)furan-2-carbohydrazide (**10b**) (0.5 g, 2.47 mmol, 1.0 equiv) in THF (10 mL). The mixture was degassed with nitrogen, refluxed for 3 h, and then stirred at room temperature overnight. Water (20 mL) was added and the mixture was extracted twice with ethyl acetate (50 mL). The combined organic phase was dried over MgSO_4_, filtered and concentrated. The crude mixture was purified by flash chromatography (ISCO system, 20 g column) using pentane/ethyl acetate (4:1 mixture) as eluent to obtain the title compound as a white solid (0.36 g, 73% yield). LC-MS (ESI) *m/z* (^35^Cl/^37^Cl) 201/203 (M+H)^+^, RT=1.77 min, 99% purity, method 1. ^1^H NMR (300 MHz, CDCl_3_) δ 7.61 (d, 1H, *J*=0.9 Hz), 7.22 (d, 1H, *J*=2.4 Hz), 6.60 (dd, 1H, *J*=2.4, 1.2 Hz), 4.98 (s, 2H).

**1-(2-Chloro-6-fluoro-phenyl)-*N*-{[5-(2-furyl)-1,3,4-thiadiazol-2-yl]methyl}methanamine (10d):** 2-Chloro-6-fluorobenzylamine (0.19 g, 1.2 mmol, 1.2 equiv), sodium iodide (0.15 g, 1.0 mmol, 1.0 equiv) and diisopropylethylamine (0.35 mL, 2.01 mmol, 2.0 equiv) were added to a solution of 2-(chloromethyl)-5-(2-furyl)-1,3,4-thiadiazole (**10c**) (200 mg, 1.0 mmol, 1.0 equiv) in acetonitrile (10 mL). The mixture was refluxed for 2 h. Water (15 mL) was added and the mixture was extracted twice with ethyl acetate (50 mL). The combined organic phase was dried over MgSO_4_, filtered and concentrated. The crude mixture was purified by flash chromatography (ISCO system, 20 g column) using pentane/ethyl acetate (3:1 mixture) as eluent to obtain the title compound as yellow oil (0.3 g, 93% yield). LC-MS (ESI) *m/z* (^35^Cl/^37^Cl) 324/326 (M+H)^+^, RT=1.98 min, 99% purity, method 1. ^1^H NMR (300 MHz, CDCl_3_) δ 7.59 (d, 1H, *J*=1.5 Hz), 7.24-7.17 (m, 2H), 7.15 (d, 1H, *J*=3.6 Hz), 7.02 (m, 1H), 6.57 (dd, 1H, *J*=3.3, 1.5 Hz), 4.22 (s, 2H), 4.09 (d, 2H, *J*=1.8 Hz), 3.99 (d, 1H, *J*=1.5 Hz).

**1-(2-Chloro-6-fluoro-phenyl)-*N*-{[5-(2-furyl)-1,3,4-thiadiazol-2-yl]methyl}-*N*-methyl-methanamine (10):** Sodium hydride (60 mg, 1.5 mmol, 1.6 equiv) was added to a solution of 1-(2-chloro-6-fluoro-phenyl)-*N*-{[5-(2-furyl)-1,3,4-thiadiazol-2-yl]methyl}methanamine (**10d**) (0.3 g, 0.926 mmol, 1.0 equiv) in DMF (5 mL) at 0°C and the mixture was stirred for 10 min. Methyl iodide (0.26 g, 1.83 mmol, 1.98 equiv) was added and stirred at room temperature for 2 h. Water (10 mL) was added and the mixture was extracted twice with ethyl acetate (25 mL). The combined organic phase was dried over MgSO_4_, filtered and concentrated. The crude mixture was purified by flash chromatography (ISCO system, 20 g column) using pentane/ethyl acetate (5:1 to 3:1 mixture) as eluent to obtain the title compound as yellow oil (0.21 g, 67% yield). LC-MS (ESI) *m/z* (^35^Cl/^37^Cl) 338/340 (M+H)^+^, RT=4.34 min, 99% purity, method 3. ^1^H NMR (400 MHz, CD_3_OD) δ 7.79 (d, 1H, *J*=1.5 Hz), 7.36-7.28 (m, 2H), 7.21 (d, 1H, *J*=2.7 Hz), 7.14-7.09 (m, 1H), 6.69 (dd, 1H, *J*=2.7, 1.5 Hz), 4.11 (s, 2H), 3.92 (s, 2H), 2.38 (s, 3H).

**2.3. Synthetic Procedure for the Preparation of Compound 21**

**2-(Chloromethyl)-5-(2-furyl)-1,3,4-oxadiazole (21a):** A suspension of phosphoryl chloride (15.8 mL, 170.29 mmol, 10.0 equiv) and *N´*-(2-chloroacetyl)furan-2-carbohydrazide (**10b**) (4.6 g, 17.03 mmol, 1.0 equiv) was heated at 90 °C under nitrogen for 4 h. Heat was removed and the solution was stirred at room temperature overnight. The excess of phosphoryl chloride was removed under vacuum and the residue was quenched with ice and then with solid sodium bicarbonate until pH=8. After 10 min water was added and the mixture was stirred for 10 min. The mixture was extracted three times with ethyl acetate. The organic phase was washed with brine, dried over Na_2_SO_4_, filtered and concentrated to give an oil that solidified upon standing. The crude mixture was purified by flash chromatography (ISCO system) eluting with hexane/ethyl acetate (4:1 to 1:1 mixture) to give the title compound as a white solid (2.56 g, 61% yield). LC-MS (ESI) *m/z* (^35^Cl/^37^Cl) 185/187 (M+H)^+^, RT=0.76 min, 97% purity, method 2. ^1^H NMR (300 MHz, DMSO-d_6_) δ 8.09 (d, 1H, *J*=1.6 Hz), 7.42 (d, 1H, *J*=4.0 Hz), 6.81 (dd, 1H, *J*=4.0, 1.6 Hz), 5.12 (s, 2H).

**2-[(2-Chloro-6-fluoro-phenyl)methoxymethyl]-5-(2-furyl)-1,3,4-oxadiazole (21):** Sodium hydride (81 mg, 2.0 mmol, 2.5 equiv) was added to a solution of (2-chloro-6-fluorophenyl)methanol (261 mg, 1.63 mmol, 2.0 equiv) in THF (10 mL). The mixture was stirred at room temperature for 30 min and then a solution of 2-(chloromethyl)-5-(furan-2-yl)-1,3,4-oxadiazole (**21a**) (150 mg, 0.813 mmol, 1.0 equiv) in THF (5 mL) was added. The mixture was stirred for 2 h. Water (20 mL) was added and the mixture was extracted three times with ethyl acetate (30 mL). The combined organic phase was washed with brine (15 mL), dried over Na_2_SO_4_, filtered and concentrated. The crude mixture was purified by flash chromatography (ISCO system, 20 g column) eluting with pentane/ethyl acetate (5:1 to 2:1 mixture) as eluent to obtain the title compound as yellow oil (70 mg, 28% yield). LC-MS (ESI) *m/z* (^35^Cl/^37^Cl) 309/311 (M+H)^+^, RT=2.01 min, 96% purity, method 1. ^1^H NMR (400 MHz, CD_3_OD) δ 7.88 (d, 1H, *J*=1.6 Hz), 7.38 (m, 1H), 7.32 (m, 1H), 7.30 (d, 1H, *J*=3.6 Hz), 7.14 (m, 1H), 6.74 (dd, 1H, *J*=3.6, 1.6 Hz), 4.88 (s, 2H), 4.78 (s, 2H).

**2.4. Synthetic Procedure for the Preparation of Compound 22**

**1-[5-(2-Furyl)-1,3,4-oxadiazol-2-yl]-*N*-methyl-methanamine (22a):** Sodium iodide (0.2 g, 1.35 mmol, 1.0 equiv) and methylamine (15 mL, 173.85 mmol, 129 equiv) were added to a solution of 2-(chloromethyl)-5-(furan-2-yl)-1,3,4-oxadiazole (**21a**) (0.25 g, 1.35 mmol, 1.0 equiv) in acetonitrile (30 mL). The mixture was stirred at 60 ºC for 30 min. Water (20 mL) was added and the mixture was extracted three times with ethyl acetate (50 mL). The combined organic phase was washed with brine (50 mL), dried over Na_2_SO_4_, filtered and concentrated. The crude mixture was purified by flash chromatography (ISCO system, 20 g column) eluting with pentane/ethyl acetate (1:1 to 1:3 mixture) to give the title compound as a yellow oil (0.22 g, 91% yield). LC-MS (ESI) *m/z* 180 (M+H)^+^, RT=1.39 min, 99% purity, method 1. ^1^H NMR (400 MHz, DMSO-d_6_) δ 8.05 (d, 1H, *J*=1.6 Hz), 7.32 (d, 1H, *J*=3.6 Hz), 6.79 (dd, 1H, *J*=3.6, 2.0 Hz), 3.92 (s, 2H), 2.31 (s, 3H).

**2-Chloro-6-fluoro-*N*-{[5-(2-furyl)-1,3,4-oxadiazol-2-yl]methy-*N*-methyl-benzamide (22):** *O*-(7-Azabenzotriazol-1-yl)-*N*,*N*,*N*',*N*'-tetramethyluronium hexafluorophosphate (611 mg, 1.61 mmol, 1.2 equiv) and triethylamine (217 mg, 2.14 mmol, 1.6 equiv) were added to a solution of 1-[5-(2-furyl)-1,3,4-oxadiazol-2-yl]-*N*-methyl-methanamine (**22a**) (0.24 g, 1.34 mmol, 1.0 equiv) and 2-chloro-6-fluorobenzoic acid (248 mg, 1.42 mmol, 1.06 equiv) in DMF (5 mL). The mixture was stirred at room temperature for 18 h. Water (20 mL) was added and the mixture was extracted three times with ethyl acetate (50 mL). The combined organic phase was washed with brine (20 mL), dried over Na_2_SO_4_, filtered and concentrated. The crude mixture was purified by flash chromatography (ISCO system, 20 g column) eluting with pentane/ethyl acetate (3:1 to 1:3 mixture) to give the title compound as a colorless oil (0.16 g, 36% yield). LC-MS (ESI) *m/z* (^35^Cl/^37^Cl) 336/338 (M+H)^+^, RT=2.76 min, 99% purity, method 3. ^1^H NMR (400 MHz, CDCl_3_) δ 7.66 (d, 1H, *J*=1.6 Hz), 7.35 (m, 1H), 7.27 (m, 1H), 7.16 (d, 1H, *J*=3.6 Hz), 7.09 (m, 1H), 6.61 (dd, 1H, *J*=3.6, 1.6 Hz), 5.21 (d, 1H, *J*=16 Hz), 5.02 (d, 1H, *J*=16 Hz), 2.99 (s, 3H).

**2.5. Synthetic Procedure for the Preparation of Compound 23**

**1-[2-(2-Chloro-6-fluoro-phenyl)ethyl]-3-(furan-2-carbonylamino)urea (23c):** 5-(2-Furyl)-3*H*-1,3,4-oxadiazol-2-one (**23a**) (286 mg, 1.88 mmol, 1.0 equiv) was added to a solution of 2-(2-chloro-6-fluoro-phenyl)ethanamine (**23b**) (488 mg, 2.82 mmol, 1.5 equiv) in ethanol (30 mL) and the solution was heated at 100 ºC for 20 h. The mixture was concentrated under vacuum and the residue was crystallized from diethyl ether to give the title compound as a solid (543 mg, 59% yield). LC-MS (ESI) *m/z* (^35^Cl/^37^Cl) 326/328 (M+H)^+^, RT=1.78 min, 96% purity, method 3.

***N*-[2-(2-Chloro-6-fluoro-phenyl)ethyl]-5-(2-furyl)-1,3,4-oxadiazol-2-amine (23d):** A mixture of 1-[2-(2-chloro-6-fluoro-phenyl)ethyl]-3-(furan-2-carbonylamino)urea (**23c**) (500 mg, 1.54 mmol, 1.0 equiv) in phosphoryl chloride (5 mL) was heated at 110 ºC for 2 h. The excess of phosphoryl chloride was removed under vacuum. The residue was quenched with a mixture of ice-water (10 mL) and pH was adjusted to 12 with a 2N aqueous solution of sodium hydroxide. The mixture was extracted twice with ethyl acetate (50 mL). The combined organic phase was washed with brine (20 mL), dried over Na_2_SO_4_, filtered and concentrated. The crude mixture was purified by flash chromatography (ISCO system, 20 g column) eluting with pentane/ethyl acetate (4:1 mixture) to give the title compound as a yellow oil (200 mg, 42% yield). LC-MS (ESI) *m/z* (^35^Cl/^37^Cl) 308/310 (M+H)^+^, RT=1.3 min, 98% purity, method 3. ^1^H NMR (400 MHz, CDCl_3_) δ 7.55 (d, 1H), 7.18 (m, 2H), 6.93 (m, 1H), 6.83 (d, 1H), 6.53 (dd, 1H), 4.89 (br s, 1H), 3.71 (q, 2H), 3.20 (t, 2H).

***N*-[2-(2-Chloro-6-fluoro-phenyl)ethyl]-5-(2-furyl)-*N*-methyl-1,3,4-oxadiazol-2-amine (23):** Sodium hydride (39 mg, 0.975 mmol, 1.5 equiv) was added to a solution of *N*-[2-(2-chloro-6-fluoro-phenyl)ethyl]-5-(2-furyl)-1,3,4-oxadiazol-2-amine (**23d**) (200 mg, 0.65 mmol, 1.0 equiv) in DMF (10 mL). The mixture was stirred for 10 min and then methyl iodide (185 mg, 1.3 mmol, 2.0 equiv) was added and stirred for 2h. The reaction was quenched with water (60 mL). The mixture was extracted three times with ethyl acetate (50 mL). The combined organic phase was dried over Na_2_SO_4_, filtered and concentrated. The crude mixture was purified by flash chromatography (ISCO system, 20 g column) eluting with ethyl ether/ethyl acetate (3:1 mixture) to give the title compound (117 mg, 56% yield). LC-MS (ESI) *m/z* (^35^Cl/^37^Cl) 322/324 (M+H)^+^, RT=4.2 min, 96% purity, method 3. ^1^H NMR (400 MHz, CD_3_OD) δ 7.62 (d, 1H), 7.05 (m, 2H), 6.92 (m, 1H), 6.83 (d, 1H), 6.54 (dd, 1H), 3.69 (t, 2H), 3.13 (t, 2H), 3.01 (s, 3H).

**3. Biological Evaluation**

**Anti-tubercular activity using butyrate as a carbon source^1^:**

*M. tuberculosis* H37Rv expressing DsRed^2, 3^ was grown for 7 days to an OD_590_ of 0.6-0.8 in Middlebrook 7H9 medium supplemented with 5 g/L BSA fraction V, 0.8 g/L NaCl, 0.05% v/v Tyloxapol, and 5 mM sodium butyrate (7H9-Ty-5BT) with 50 µg/mL hygromycin. Cultures were filtered, adjusted to a theoretical OD_590_ of 0.030 in 7H9-Ty-BT with 10 mM sodium butyrate and used to inoculate 96-well plates containing 2-fold serial dilutions of compounds. Plates were incubated at 37 °C for 6 days, and growth was measured by OD_590_ and fluorescence (Ex 560 nm; Em 590 nm). The MIC was determined using a 4 parameter curve fit.

**Anti-tubercular activity using glucose as a carbon source^4^:**

*M. tuberculosis* H37Rv expressing DsRed was grown to late log phase (OD_590_ of 0.6-1.0) in Middlebrook 7H9 medium supplemented with 0.05% w/v Tween 80, 10% v/v oleic acid, albumin, dextrose and catalase supplement (OADC; Becton Dickinson) (7H9-Tw-OADC and 50 µg/mL hygromycin. Cultures were filtered, adjusted to a theoretical OD_590_ of 0.020 in 7H9-Tw-OADC and used to inoculate 96-well plates containing 2-fold serial dilutions of compounds. Plates were incubated at 37 °C for 5 days. Growth was measured by OD_590_ and fluorescence (Ex 560 nm; Em 590 nm). The MIC was determined using a 4 parameter curve fit.

**Cytotoxicity:**

Vero cells (ATCC^®^ CCL81) were grown in DMEM, high glucose, GlutaMAX™ (Invitrogen), 10% FBS, 50 units/mL penicillin, and 50 µg/mL streptomycin, seeded as 1200 cells per well in 384-well plates and incubated for 24 h at 37 °C with 5% CO_2._ Compounds were added as a 3-fold serial dilution series and cells incubated for 2 days at 37 °C with 5% CO_2._ Viability was measured using CellTiter-Glo® Reagent (Promega). The compound concentration that resulted in 50% inhibition was calculated using a 4 parameter curve fit.^5, 6^

**4. Method for microsomal stability**:

To determine microsomal turnover, test compounds were incubated (2 μM) with liver microsomes in the presence of NADPH, and loss of parent molecule was measured by LC-MS after 30 min.

**5. Method for PK studies**:

**In Vivo Pharmacokinetic Studies**. Compounds 1, 2 and 3 were administered intravenously at 1 mg/kg (1 ml/kg) in Dimethyl acetamide 25%v/v, ethanol 15%v/v, propylene glycol 10%v/v, 2 pyrrolidone 25%v/v, PW 25%v/v in fed animal, or orally at 10 mg/kg (10 ml/kg) in Hydroxyethylcellulose 1% w/v/polysorbate 80 0.25% v/v/Antifoam 1510-US 0.05% v/v/PW (probe sonicate), in fasted animals, respectively. Additional study was conducted for compound 2 wherein the animals were dosed 1-aminobenzotriazole (100 mpk) intraperitoneally one hour prior to dosing compound 2 either intravenously (fed animals) at 1 mg/kg or orally (fasted animals) at 10 mg/kg. Three animals were dosed per study arm.

Serial blood samples were collected at 0.08, 0.25, 0.75, 2, 4, 8, 24 for intravenous and 0.25, 0.5, 1, 2, 4, 8, 24 for oral administration. The blood was collected via a tail snip directly into a 20-μL EDTA-coated capillary and immediately spotted onto a Whatman DMPK-C dried blood sample (DBS) card and analysed using LC-MS/MS, as described previously.^7^ Noncompartmental pharmacokinetic parameters were calculated using Watson version 7.4 (Thermo Scientific, Waltham, MA).

Figure s1

a


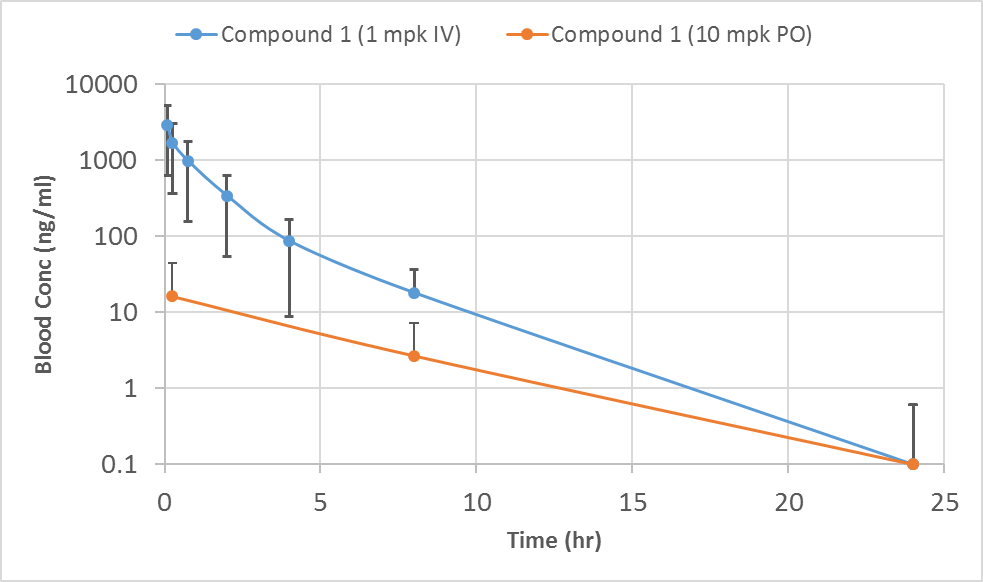


b


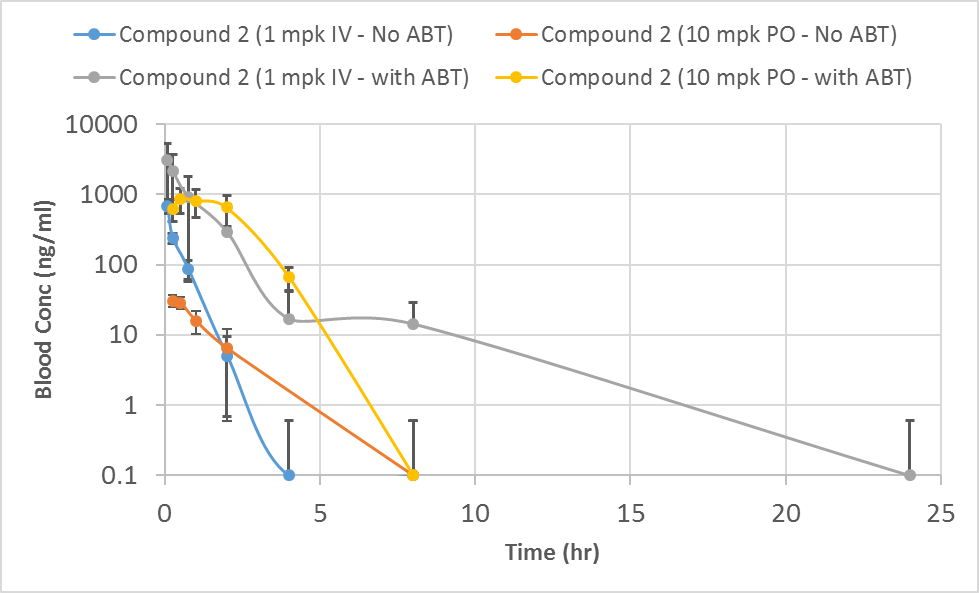


c


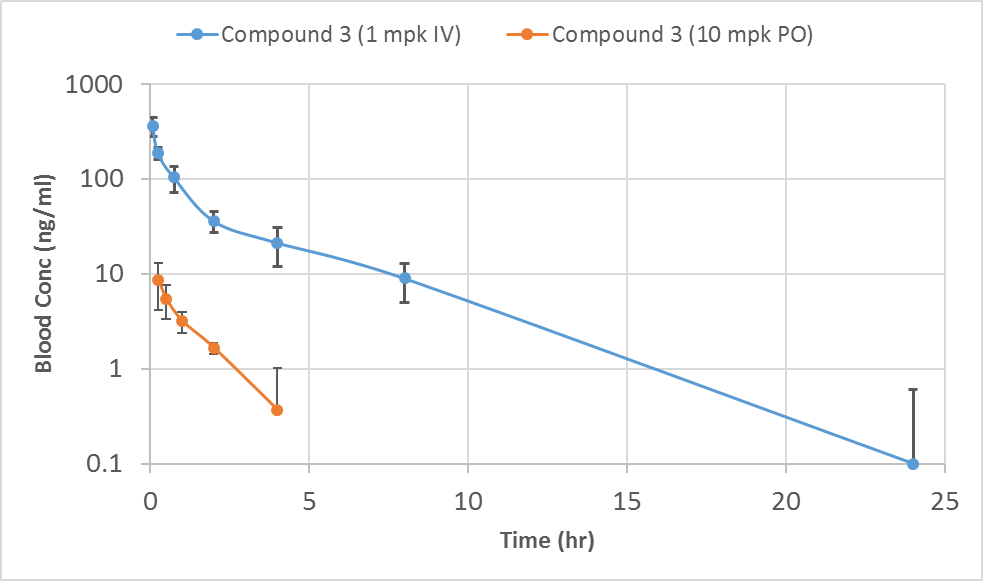


Figure s1: Mouse mean blood concentration vs. time profiles for compounds 1, 2 and 3. (a) Compound 1 (b) Compound 2 with and without pre-treatment with ABT (c) Compound 3.

**Fraction unbound in mouse plasma:^8^**

| **Compound ID** | **Unbound fraction in plasma** |
| --- | --- |
| **1** | **0.011** |
| **2** | **0.123** |
| **3** | **<0.015** |

**References**

1. Early JV, Casey A, Martinez-Grau MA, et al. Oxadiazoles Have Butyrate-Specific Conditional Activity against Mycobacterium tuberculosis. *Antimicrobial agents and chemotherapy.* 2016;60(6): 3608-3616.

2. Carroll P, Schreuder LJ, Muwanguzi-Karugaba J, et al. Sensitive detection of gene expression in mycobacteria under replicating and non-replicating conditions using optimized far-red reporters. *PloS one.* 2010;5(3): e9823.

3. Zelmer A, Carroll P, Andreu N, et al. A new in vivo model to test anti-tuberculosis drugs using fluorescence imaging. *The Journal of antimicrobial chemotherapy.* 2012;67(8): 1948-1960.

4. Ollinger J, Bailey MA, Moraski GC, et al. A dual read-out assay to evaluate the potency of compounds active against Mycobacterium tuberculosis. *PloS one.* 2013;8(4): e60531.

5. Crouch SP, Kozlowski R, Slater KJ, Fletcher J. The use of ATP bioluminescence as a measure of cell proliferation and cytotoxicity. *Journal of immunological methods.* 1993;160(1): 81-88.

6. Peternel L, Kotnik M, Prezelj A, Urleb U. Comparison of 3 cytotoxicity screening assays and their application to the selection of novel antibacterial hits. *Journal of biomolecular screening.* 2009;14(2): 142-150.

7. Wickremsinhe ER, Perkins EJ. Using dried blood spot sampling to improve data quality and reduce animal use in mouse pharmacokinetic studies. *J Am Assoc Lab Anim Sci.* 2015;54(2): 139-144.

8. Zamek-Gliszczynski MJ, Sprague KE, Espada A, et al. How well do lipophilicity parameters, MEEKC microemulsion capacity factor, and plasma protein binding predict CNS tissue binding? *J Pharm Sci.* 2012;101(5): 1932-1940.
